# Impact of Pulmonary Rehabilitation on Quality of Life in Patients With Chronic Obstructive Pulmonary Disease: A Prospective Observational Cohort Study

**DOI:** 10.7759/cureus.111476

**Published:** 2026-06-25

**Authors:** Vijay K Goad, Deepak Makode, Harshita G Pawar, Mahendra K Bharti

**Affiliations:** 1 General Practice, Nandkumar Singh Chouhan Government Medical College, Khandwa, IND; 2 General Practice, All India Institute of Medical Sciences, Bhopal, IND; 3 General Practice, Ram Krishna Medical College Hospital and Research Centre, Bhopal, IND; 4 Pulmonary Medicine, Government Medical College, Datia, IND

**Keywords:** cat score, copd, gold, pulmonary rehabilitation, st. george’s respiratory questionnaire-c

## Abstract

Background: Chronic obstructive pulmonary disease (COPD) is a long-term respiratory disorder characterized by persistent airflow limitation due to structural changes in the airways and lung parenchyma, leading to progressive symptoms and functional impairment. This study aimed to evaluate the association of pulmonary rehabilitation (PR) with health-related quality of life (QoL), symptom burden, and functional status among patients with COPD.

Methods: A prospective observational cohort study was conducted among patients with COPD attending the outpatient department of Government Medical College, Datia. Participants receiving PR as part of routine clinical management and providing informed consent were enrolled and followed prospectively. Diagnosis was confirmed through clinical evaluation and spirometry, and patients were classified according to the Global Initiative for Chronic Obstructive Lung Disease (GOLD) 2023 categories (A, B, and E). Baseline assessments included the St. George's Respiratory Questionnaire (SGRQ) for COPD patients, the COPD Assessment Test (CAT), and pulmonary function tests. Patients participated in an eight-week supervised PR program conducted twice weekly, followed by continuation of home-based exercises for at least five days per week. Follow-up assessments were performed six months after enrollment. Data were analyzed using the Statistical Package for the Social Sciences software, version 26 (IBM Corp., Armonk, NY).

Results: A total of 107 participants were included, with a mean age of 62.5 ± 6.2 years. Most were male participants (82, 76.6%). GOLD Category E accounted for 69 (64.5%) participants and Category B for 38 (35.5%). Moderate airflow limitation (GOLD Grade 2) was present in 76 (71.0%) participants. At six-month follow-up, significant improvements were observed in QoL measures, including the SGRQ total score (p < 0.001) and all SGRQ domains (p < 0.001). CAT scores decreased significantly (p < 0.001). No statistically significant change was observed in forced expiratory volume in one second (% predicted) (p = 0.067).

Conclusion: Participation in PR was associated with significant improvements in QoL, symptom burden, and functional status among patients with COPD, despite no significant changes in spirometric parameters. These findings support the integration of PR into comprehensive COPD care. Further multicentric studies with control groups and longer follow-up are needed to evaluate long-term effectiveness and identify optimal rehabilitation strategies.

## Introduction

Chronic obstructive pulmonary disease (COPD) is a chronic respiratory condition characterized by irreversible or partially reversible airflow obstruction resulting from airway and alveolar abnormalities. The disease commonly presents with breathlessness, chronic cough, sputum production, and recurrent exacerbations [1].

COPD is a major contributor to global morbidity and mortality and ranks among the leading causes of death worldwide. A substantial proportion of COPD-related deaths occur in low- and middle-income countries, accounting for nearly 90% of the global burden. It is estimated that approximately three million deaths were attributable to COPD in 2012, representing about 6% of total global mortality [1]. Despite its high burden, COPD is largely preventable and manageable with appropriate interventions.

Beyond its pulmonary manifestations, COPD is increasingly recognized as a systemic condition with effects on multiple organ systems. Smoking cessation remains the most effective intervention to slow the decline in lung function. However, additional therapeutic strategies are essential to improve functional capacity and enhance quality of life (QoL). Comprehensive management of COPD involves a combination of pharmacological treatment, pulmonary rehabilitation (PR), acute care, and palliative support.

PR is a structured multidisciplinary intervention aimed at improving physical performance, symptom control, and psychological well-being in individuals with chronic respiratory diseases. The program generally includes supervised exercise training, breathing exercises, education, nutritional counseling, and self-management strategies tailored to patient needs. When integrated with medical therapy, oxygen optimization, and smoking cessation, PR is considered one of the most effective approaches for managing symptomatic COPD [2].

Exercise training is a fundamental component of PR, contributing to improvements in muscle strength, endurance, and patient confidence, while also alleviating dyspnea. These benefits facilitate better participation in daily activities and reduce healthcare utilization [3].

Evidence consistently demonstrates that PR enhances exercise tolerance, reduces symptom burden, and improves health-related QoL across all stages of COPD severity. Patients with advanced disease, particularly those with chronic hypercapnic respiratory failure, may derive the greatest benefit [4]. Optimal outcomes are generally achieved with rehabilitation programs lasting six to eight weeks, with no clear additional benefit observed with longer durations such as 12 weeks. Standard protocols recommend supervised sessions at least twice weekly, incorporating aerobic activities (e.g., walking), resistance training, interval training, and exercises targeting both upper and lower limbs. Additional modalities such as inspiratory muscle training, neuromuscular electrical stimulation, and flexibility exercises may also be included [5].

Although PR is well established as an effective intervention for improving symptoms and QoL in COPD patients, there is limited context-specific evidence evaluating its impact using standardized patient-reported outcome measures in local populations. Therefore, the present study was undertaken to assess the effect of PR on QoL, symptom burden, and functional status among patients with COPD using validated assessment tools.

## Materials and methods

Study design and setting

This single-arm prospective observational cohort study was conducted in the Department of Pulmonary Medicine at Government Medical College, Datia, India. The study prospectively followed patients with COPD who participated in PR as part of routine clinical management and evaluated changes in QoL, symptom burden, and functional status over time. Consecutive eligible patients were recruited during the study period and followed individually from the time of enrollment. PR was conducted over eight weeks, and follow-up assessments were performed six months after enrollment.

Study duration

Participants were recruited between April 2024 and June 2024 and followed for six months after enrollment. The final follow-up assessments were completed in December 2024.

Study population

Consecutive patients attending the outpatient department who were clinically diagnosed with COPD and were advised PR as part of standard care were screened for eligibility.

Inclusion criteria

Patients diagnosed with COPD according to the Global Initiative for Chronic Obstructive Lung Disease (GOLD) 2023 guidelines and classified into GOLD groups B or E were eligible for inclusion [1]. Only clinically stable patients without evidence of an acute exacerbation within the preceding four weeks, who were willing to participate in PR and had provided written informed consent, were enrolled.

Exclusion criteria

Patients with acute COPD exacerbation at enrollment or within the preceding four weeks, uncontrolled heart failure, severe lower limb arthritis, or neurological disorders limiting participation in exercise training were excluded from the study.

Sample size calculation

The sample size was calculated based on the study by McCarthy et al. [6], which reported that 53% of patients achieved a clinically significant improvement (>4 units) in the St. George's Respiratory Questionnaire (SGRQ) following PR.

The minimum sample size was estimated to be 96 participants using the following formula:

$n = \frac{Z^{2} \times p \times q}{d^{2}}$

where p = 0.53, q = 0.47, Z = 1.96 at 95% confidence level, and d = 0.10. After accounting for a 10% nonresponse rate, the final required sample size was calculated as 107 participants.

Baseline assessment and diagnosis

The diagnosis of COPD was confirmed using clinical history, physical examination, and postbronchodilator spirometry according to GOLD 2023 recommendations. Baseline assessment included demographic characteristics, smoking history, occupational exposure, modified Medical Research Council (mMRC) dyspnea grading, COPD Assessment Test (CAT) score, SGRQ score, and pulmonary function testing, including postbronchodilator forced expiratory volume in one second (FEV1) (% predicted).

Written permission for use of the SGRQ was obtained from the SGRQ Office, City St George's, University of London.

PR program

Participants receiving PR as part of routine clinical care were followed prospectively. The PR program was conducted for a duration of eight weeks and was supervised by the pulmonary medicine team, including pulmonologists and trained physiotherapists. The program included respiratory muscle training such as pursed-lip breathing, diaphragmatic breathing, and deep breathing exercises; upper and lower limb stretching and strengthening exercises; aerobic exercise training; patient education; nutritional counseling; and psychological support. Participants attended supervised sessions twice weekly at the hospital for eight weeks and were subsequently advised to continue the prescribed home-based exercise program for at least five days per week until the six-month follow-up assessment.

Follow-up assessment

Follow-up assessment was conducted at six months using the SGRQ, CAT, mMRC dyspnea scale, and postbronchodilator pulmonary function testing. Arterial blood gas analysis was not repeated during follow-up, as it was used only for baseline clinical assessment and was not a predefined outcome measure.

Study tools

The assessment tools used in the study included the SGRQ, CAT, and mMRC dyspnea scale.

St. George's Respiratory Questionnaire

The SGRQ is a validated disease-specific instrument used to assess health-related QoL in patients with COPD. It evaluates symptoms, activity limitation, and psychosocial impact associated with respiratory disease. Scores range from 0 to 100, with higher scores indicating poorer health status. A reduction of four or more points is considered clinically meaningful. Written permission for use of the questionnaire was obtained from the SGRQ Office, City St George’s, University of London [7].

COPD Assessment Test

The CAT is an eight-item validated questionnaire designed to measure the impact of COPD on daily functioning and overall health status. Each item is scored from 0 to 5, producing a total score ranging from 0 to 40. Higher scores indicate greater symptom burden and disease impact [8].

The mMRC Dyspnea Scale

The mMRC dyspnea scale was used to assess the severity of breathlessness. The scale grades dyspnea from 0 to 4 according to the degree of physical activity associated with respiratory discomfort, with higher grades indicating greater functional limitation [9].

Statistical analysis

Data were entered into Microsoft Excel version 2016 (Microsoft Corporation, Redmond, WA) and analyzed using IBM Statistical Package for the Social Sciences Statistics, version 26 (IBM Corp., Armonk, NY). Continuous variables were expressed as mean ± standard deviation, while categorical variables were expressed as frequencies and percentages. Changes between baseline and follow-up measurements were assessed using paired t-tests. p < 0.05 was considered statistically significant at the 95% confidence level. As all participants received PR and no comparison group was included, measures of association such as risk ratios were not calculated.

Ethical considerations

The study was approved by the Institutional Ethics Committee, Government Medical College, Datia (approval no. 129/RESPI/GMC/IECBMHR/2024). Written informed consent was obtained from all participants prior to enrollment.

## Results

A total of 107 patients were included in the study. The mean age of participants was 62.5 ± 6.2 years, with ages ranging from 54 to 75 years, indicating that the study population predominantly consisted of older adults. The study population was predominantly male, with 82 (76.6%) men and 25 (23.4%) women. Regarding smoking status, 41 (38.3%) were former smokers, 32 (30.0%) were active smokers, 24 (22.4%) were never smokers, and 10 (9.3%) were passive smokers (Table 1).

**Table 1:** Gender and smoking pattern of study participants (n = 107) Gender is categorized as male and female. Smoking status is classified as never smokers (lifetime nonsmokers), former smokers (previous smokers who have quit), active smokers (current smokers), and passive smokers (nonsmokers exposed to environmental tobacco smoke). Total represents the overall study population (n = 107)

Variable	Category	Frequency (n)	Percentage (%)
Gender	Male	82	76.6
	Female	25	23.4
Smoking status	Never smokers	24	22.4
	Former smokers	41	38.3
	Active smokers	32	30.0
	Passive smokers	10	9.3
Total	-	107	100

Occupational exposure to smoke was the most common, reported by 45 (42.0%) participants, followed by dust exposure by 31 (29.0%). Twenty-eight (26.2%) participants reported no occupational exposure, while only three (2.8%) were exposed to toxic fumes. The predominance of smoke and dust exposure suggests that occupational and environmental factors may have contributed substantially to the development and progression of COPD in this study population.

Regarding disease severity, most participants had GOLD grade 2 (moderate) disease (76 (71.0%)), followed by GOLD grade 3 (severe; 22 (20.6%) and GOLD grade 1 (mild; 9 (8.4%)). Based on the combined assessment, most participants belonged to GOLD category E (69 (64.5%)), while 38 (35.5%) were categorized as belonging to category B. Assessment of dyspnea using the mMRC scale revealed that 66 (61.7%) participants had Grade 2 dyspnea, followed by 31 (29.0%) with Grade 1 and 10 (9.3%) with Grade 3 (Table 2).

**Table 2:** Occupational exposure and clinical profile of study participants (n = 107) Occupational exposure is categorized as no exposure, dust, smoke, and toxic fumes GOLD grading represents the severity of airflow limitation based on FEV1% predicted: GOLD 1 (mild), GOLD 2 (moderate), GOLD 3 (severe), and GOLD 4 (very severe) GOLD category (A, B, E) reflects the combined assessment of symptom burden and exacerbation risk mMRC: dyspnea severity is graded on a scale from 0, indicating breathlessness only during strenuous physical activity, to 4, where the individual experiences breathlessness severe enough to prevent leaving the house. Total represents the overall study population (n = 107) GOLD: Global Initiative for Chronic Obstructive Lung Disease; mMRC: modified Medical Research Council; FEV1: forced expiratory volume in one second

Variable	Category	Frequency (n)	Percentage (%)
Occupational exposure	No exposure	28	26.2
	Dust	31	29
	Toxic fumes	3	2.8
	Smoke	45	42
GOLD grade	GOLD 1 (mild)	9	8.4
	GOLD 2 (moderate)	76	71
	GOLD 3 (severe)	22	20.6
GOLD category	B	38	35.5
	E	69	64.5
mMRC grade	1	31	29
	2	66	61.7
	3	10	9.3

Following PR, there was a statistically significant improvement in the SGRQ total score (95% CI: 1.62-3.68; p < 0.001) and all its domains: symptoms (95% CI: 7.10-9.02; p < 0.001), activity (95% CI: 9.89-12.05; p < 0.001), and impact (95% CI: 8.72-10.58; p < 0.001). The CAT score also showed a significant reduction (95% CI: 1.45-1.85; p < 0.001). However, no statistically significant change was observed in FEV1 (% predicted) (95% CI: -1.15 to 0.03; p = 0.067), indicating that PR improved QoL and functional status without significant changes in spirometric parameters (Table 3).

**Table 3:** Comparison of clinical parameters before and after pulmonary rehabilitation Mean values are presented for prepulmonary (before) and postpulmonary (after) rehabilitation. Mean difference represents the change between before and after values. 95% CI indicates the precision of the mean difference. t value is derived from a paired t-test ^*^p value <0.05 is considered statistically significant df: degrees of freedom; SD: standard deviation; CI: confidence interval; SGRQ: St. George's Respiratory Questionnaire; CAT: Chronic obstructive pulmonary disease Assessment Test; FEV1: forced expiratory volume in one second

Parameter	Time	Mean	SD	95% CI (lower-upper)	t value	df	p value	
								SGRQ total	Before	53.65	5.39	1.62-3.68	5.1	106	<0.001^*^	
After	51	6.46													
SGRQ symptoms	Before	52.68	5.22	7.10-9.02	16.2	106	<0.001^*^	
	After	44.62	6.57					
SGRQ activity	Before	53.74	5.11	9.89-12.05	20.05	106	<0.001^*^	
	After	42.76	6.65					
SGRQ impact	Before	52.91	4.74	8.72-10.58	22.1	106	<0.001^*^	
	After	43.26	6.35					
CAT score	Before	18.76	2.98	1.45-1.85	15.9	106	<0.001^*^	
	After	17.12	3.11					
FEV1 (%)	Before	66.62	14.96	-1.15 to 0.03	-1.85	106	0.067	
	After	67.21	14.04					

## Discussion

This study demonstrated significant improvement in QoL and symptom burden among patients with COPD after PR. Reduced SGRQ and CAT scores after rehabilitation indicated improvement in respiratory symptoms, daily functioning, and overall health-related QoL. Although a majority of participants belonged to GOLD Category E, all were clinically stable at recruitment. GOLD Category E classification was based on prior exacerbation history and symptom burden rather than the presence of an active exacerbation. Although the reduction in total SGRQ score was statistically significant, it did not reach the conventional minimal clinically important difference of four points. Nevertheless, significant improvements were observed across all SGRQ domains and CAT scores, suggesting meaningful benefits in patient-reported outcomes.

The findings of this study are comparable to those reported by Kanner et al., who observed improvements in respiratory symptoms and functional outcomes following structured intervention programs in patients with COPD [10]. Similarly, Scanlon et al. demonstrated that rehabilitation and supportive management strategies contributed to better clinical outcomes and symptom control in patients with mild-to-moderate COPD [11].

Makris et al. reported significant improvement in exercise tolerance, dyspnea severity, and activities of daily living after PR [12]. This study also demonstrated improved functional status and patient-reported outcomes after rehabilitation. Lacasse et al. observed clinically meaningful improvement in QoL measures, including SGRQ and CAT scores, after PR [13]. A similar reduction in SGRQ and CAT scores was observed in this study, indicating improved health-related QoL and symptom burden.

Kumar et al. demonstrated improvements in CAT scores, exercise capacity, and dyspnea severity among COPD patients undergoing supervised PR in a tertiary care setting [14]. These findings are consistent with this study, which also showed improved symptom control and functional performance after rehabilitation. Panwar et al. reported significant enhancement in QoL and functional exercise capacity after PR [15]. The comparable improvement in SGRQ scores in this study further supports the beneficial role of rehabilitation programs in enhancing patient well-being.

Singh et al. emphasized the utility of home-based PR strategies in improving accessibility and patient participation, particularly in rural settings [16]. In this study, the continuation of home-based exercises after supervised sessions may have contributed to sustained clinical improvement. Amin et al. demonstrated that disease severity is strongly associated with QoL and functional exercise capacity [17]. Similarly, this study observed that patients with greater baseline impairment experienced meaningful improvement after rehabilitation.

Sepulveda Loyola et al. reported that incorporating balance training into PR programs improves physical performance and functional outcomes in patients with COPD [18]. Although balance training was not assessed separately in this study, overall improvement in functional status supports the value of comprehensive rehabilitation strategies. Yadav et al. highlighted the influence of environmental and lifestyle factors on COPD severity and functional limitation [19]. These observations reinforce the need for individualized and multidisciplinary rehabilitation approaches tailored to patient-specific risk factors and disease burden.

Despite significant improvements in QoL measures and functional outcomes, no statistically significant change in FEV1 was observed in this study. Similar findings have been reported in previous studies, suggesting that PR primarily improves symptom control, exercise tolerance, and patient-reported outcomes rather than spirometric parameters [4,5].

Limitations

This study was conducted at a single tertiary care center with a relatively small sample size (n = 107), which may limit the generalizability of the findings. The observational pre-post design without a control group, randomization, or blinding limits causal inference. The six-month follow-up period may not adequately assess long-term outcomes such as exacerbations, hospitalizations, and mortality. Adherence to the home-based rehabilitation program was based on self-report and was not objectively monitored. Patient-reported outcome measures, including the SGRQ and CAT, may be subject to reporting bias. Potential confounding factors, such as socioeconomic status, nutritional status, environmental exposures, and comorbidities, were not controlled for. Additionally, subgroup, correlation, and regression analyses were not performed; therefore, predictors of response to PR could not be identified. Further multicentric studies with larger sample sizes and longer follow-up are needed to validate these findings. The study employed a single-arm observational cohort design without a comparison group. Consequently, measures of association such as risk ratios could not be calculated, limiting the ability to quantify the relative effect of PR compared with patients not receiving rehabilitation.

## Conclusions

Participation in PR was associated with significant improvements in QoL and symptom burden in patients with COPD and should be incorporated into routine disease management strategies. Although spirometric improvement was limited, meaningful enhancement in functional status and patient-reported outcomes was observed. Further multicentric studies with longer follow-up periods are recommended to evaluate long-term effectiveness and identify optimal rehabilitation protocols.
